# Supplementary material for: Comparative reassessment of AcrB efflux inhibitors reveals differential impact of specific pump mutations on the activity of potent compounds
Source: Microbiol Spectr. 2024 Jan 3;12(2):e03045-23. doi: 10.1128/spectrum.03045-23 (PMC10846202; doi:10.1128/spectrum.03045-23)
Supplement: Table S1 to S4, Fig. S1 and S2 — Tables S1 (MICs of drugs alone and fold-MIC decreases with EPIs), S2 (Conformational distance changes in AcrB), S3 (Atoms used for distance measurements), and S4 (HPLC_MS parameters for linezolid measurement) and Fig. S1 (acrB gene expression data) and S2 (Growth curves of strains). [file spectrum.03045-23-s0001.pdf]

SUPPLEMENTAL MATERIAL

Comparative reassessment of AcrB efflux inhibitors reveals differential impact of specific pump mutations on the activity of potent compounds.

Sabine Schuster[a]*, Martina Vavra[a], Dave A. N. Wirth[a], and Winfried V. Kern[a,b]

[a] *Division of Infectious Diseases, Department of Medicine II, University Hospital and Medical Center, Freiburg, Germany;* [b] *Faculty of Medicine, Albert-Ludwigs-University, Freiburg, Germany*

Table S1. Drug sensitizing activity of EPIs

	Drug[a]									
***E. coli* strain/AcrB mutant**	LVX	MXF	LZD	CLI	OXA	CXM	NOV	MIN	RIX	AZM
**3-AG100**										
MIC [µg/ml][b] drug alone	2 (± 0)	4 (± 0)	912 (± 20)	856 (± 74)	531 (± 17)	28 (± 1)	589 (± 36)	3 (± 0)	29 (± 2)	280 (± 11)
	**fold MIC decrease[b] with EPI[c]**									
48 µM PAβN	4 (± 0)	9 (± 1.1)	12 (± 1)	16 (± 3.8)	7 (± 0.8)	1 (± 0.2)	137 (± 17)	10 (± 0.9)	69 (± 17)	21 (± 2.3)
440 µM NMP	8 (± 0.4)	7 (± 0.5)	31 (± 1.7)	12 (± 2.8)	6 (± 0.4)	4 (± 0.4)	6 (± 0.7)	9 (± 0.7)	2 (± 0.3)	5 (± 0.6)
50 µM Mefloquine	2 (± 0.4)	2 (± 0.4)	1 (± 0)	2 (± 0.4)	1 (± 0)	1 (± 0)	1 (± 0)	1 (± 0)	1 (± 0)	1 (± 0)
670 µM Artesunate	1 (± 0)	2 (± 0.4)	1 (± 0)	1 (± 0)	1 (± 0)	1 (± 0)	1 (± 0)	1 (± 0.2)	2 (± 0.4)	3 (± 0.7)
100 µM Sertraline	4 (± 1)	2 (± 0.4)	4 (± 0.4)	12 (± 5.1)	4 (± 0)	4 (± 1.1)	1 (± 0.3)	4 (± 0.4)	2 (± 0.2)	2 (± 0.4)
200 µM Pimozide	2 (± 0)	2 (± 0.4)	2 (± 0.4)	2 (± 0.4)	2 (± 0.4)	2 (± 0.4)	1 (± 0)	1 (± 0)	1 (± 0.2)	1 (± 0)
25 µM MBX2319	7 (± 0.6)	5 (± 0.8)	3 (± 0.3)	8 (± 3.9)	4 (± 0.4)	7 (± 0.6)	2 (± 0.4)	4 (± 0.6)	1 (± 0.2)	4 (± 0.4)
130 µM Lanatosid C	1 (± 0)	1 (± 0.4)	2 (± 0)	1 (± 0)	1 (± 0.1)	1 (± 0.1)	1 (± 0.8)	1 (± 0.4)	1 (± 0.1)	1 (± 0.3)
503 µM Daidzein	1 (± 0)	1 (± 0)	1 (± 0)	1 (± 0)	1 (± 0.1)	1 (± 0.4)	1 (± 0.5)	1 (± 0.1)	1 (± 0)	1 (± 0.3)
330 µM NDGA	2 (± 0.3)	1 (± 0.1)	2 (± 0.2)	8 (± 1.3)	4 (± 0.8)	4 (± 0.9)	3 (± 0.4)	1 (± 0.2)	1 (± 0.1)	15 (± 9.1)
240 µM Mangiferin	1 (± 0.1)	1 (± 0.2)	1 (± 0.3)	1 (± 0)	1 (± 0)	1 (± 0.1)	1 (± 0.1)	1 (± 0)	1 (± 0.2)	1 (± 0.3)
170 µM Plumbagine	1 (± 0.2)	1 (± 0.2)	1 (± 0)	2 (± 0)	1 (± 0)	1 (± 0.2)	1 (± 0.2)	1 (± 0.2)	1 (± 0.2)	2 (± 0)
350 µM Shikonin	1 (± 0)	1 (± 0)	1 (± 0.2)	1 (± 0)	1 (± 0)	1 (± 0.2)	1 (± 0.2)	1 (± 0.2)	1 (± 0.2)	2 (± 0.4)
400 µM Quercetin	1 (± 0.2)	1 (± 0)	1 (± 0)	2 (± 0.6)	1 (± 0)	1 (± 0)	1 (± 0.2)	1 (± 0.3)	1 (± 0.1)	1 (± 0)
25 µM MBX2931	17 (± 2.9)	11 (± 1.5)	8 (± 0)	6 (± 1)	11 (± 2.2)	13 (± 1.5)	5 (± 0.6)	7 (± 1.8)	3 (± 0.4)	19 (± 4)
12.5 µM MBX3132	23 (± 2.6)	25 (± 5.1)	28 (± 2.2)	30 (± 4.7)	38 (± 8.5)	60 (± 3.4)	37 (± 6.7)	20 (± 5.6)	6 (± 0.7)	46 (± 6.9)
12.5 µM MBX3135	23 (± 2.6)	27 (± 5)	25 (± 2.7)	38 (± 5.7)	43 (± 8.5)	53 (± 5)	43 (± 6.7)	15 (± 3.2)	6 (± 0.8)	37 (± 12.2)
12.5 µM MBX3796	17 (± 2.9)	17 (± 2.9)	23 (± 4)	29 (± 2.4)	32 (± 0)	40 (± 7.3)	27 (± 3.1)	16 (± 3.3)	5 (± 0.9)	53 (± 6.2)
50 µM BM-19	3 (± 0.3)	5 (± 0.5)	7 (± 0.7)	4 (± 0.3)	2 (0.2)	1 (± 0.2)	6 (± 1.6)	4 (± 0.3)	8 (± 1.3)	2 (± 0.4)
220 µM Procyanidin A2	1 (± 0)	1 (± 0.1)	1 (± 0.3)	2 (± 0.7)	2 (0.6)	11 (± 8.4)	1 (± 0)	1 (± 0)	1 (± 0)	1 (± 0)
200 µM Reserpine	1 (± 0.3)	1 (± 0.2)	1 (± 0.3)	1 (± 0)	1 (± 0)	1 (± 0)	1 (± 0)	1 (± 0)	1 (± 0)	1 (± 0.1)
80 µM Domperidone	1 (± 0.3)	1 (± 0.3)	1 (± 0)	1 (± 0)	1 (± 0)	1 (± 0.4)	1 (± 0.1)	1 (± 0)	1 (± 0)	1 (± 0.1)
480 µM t-Cinnamaldehyde	1 (± 0)	1 (± 0)	1 (± 0.2)	1 (± 0.2)	1 (± 0)	1 (± 0.2)	1 (± 0.1)	1 (± 0)	1 (± 0)	1 (± 0)
460 µM Amitriptyline	2 (± 0.3)	1 (± 0.3)	2 (± 0)	2 (± 0)	2 (± 0)	2 (± 0.7)	2 (± 0.3)	1 (± 0.3)	2 (± 0.3)	1 (± 0.3)
200 µM Chlorpromazine	2 (± 0)	1 (± 0)	1 (± 0.3)	2 (± 0)	2 (± 0)	2 (± 0.3)	1 (± 0.2)	2 (± 0)	1 (± 0)	1 (± 0.3)
330 µM Dihydrocapsaicin	2 (± 0.4)	1 (± 0.2)	1 (± 0.2)	1 (± 0)	1 (± 0)	1 (± 0.5)	1 (± 0.2)	1 (± 0)	1 (± 0)	1 (± 0)
530 µM 1-Benzyl-1,4-diazepane	2 (± 0.3)	1 (± 0.3)	1 (± 0.3)	2 (± 0)	1 (± 0)	1 (± 0.1)	1 (± 0.1)	1 (± 0.3)	1 (± 0.1)	3 (± 0.5)
50 µM NA #15	2 (± 0)	2 (± 0.3)	3 (± 0.5)	2 (± 0)	2 (± 0)	1 (± 0.3)	2 (± 0.8)	1 (± 0)	1 (± 0.3)	5 (± 1.1)
50 µM NA #17	5 (± 1.4)	7 (± 3.6)	9 (± 2.9)	4 (± 0)	3 (± 0.5)	25 (± 16)	65 (± 30)	2 (± 0)	17 (± 7.1)	43 (± 8.7)
25 µM NA #18	2 (± 0.4)	2 (± 0.2)	7 (± 0.7)	2 (± 0)	1 (± 0.2)	2 (± 0.2)	1 (± 0)	2 (± 0)	8 (± 2.8)	21 (± 4.3)
50 µM NA #20	7 (± 0.9)	6 (± 1.3)	11 (± 2.6)	6 (± 1)	28 (± 3.5)	28 (± 12)	64 (± 23)	3 (± 0.5)	24 (± 4)	18 (± 7)
50 µM NA #21	2 (± 0)	2 (± 0.3)	3 (± 0.5)	2 (± 0)	2 (± 0)	1 (± 0.3)	1 (± 0.3)	1 (± 0.3)	1 (± 0)	2 (± 0.3)
100 µM BDM88855	12 (±2.8)	16 (± 0)	32 (± 0)	16 (± 0)	21 (± 4.4)	32 (± 0)	16 (± 0)	16 (± 0)	4 (± 0)	32 (± 0)
509 µM PCPP	9 (± 1.8)	6 (± 0.9)	6 (± 0.9)	4 (± 0.4)	4 (± 0)	1 (± 0.2)	4 (± 0)	6 (± 0.9)	1 (± 0.2)	8 (± 0)
587 µM RP1	2 (± 0.3)	1 (± 0)	2 (± 0.2)	1 (± 0)	1 (± 0.2)	2 (± 0.6)	1 (± 0.3)	1 (± 0.2)	1 (± 0.1)	2 (± 0.3)
340 µM Proguanil	6 (± 1.5)	12 (± 1.6)	14 (± 1.2)	11 (± 1.6)	8 (± 1.2)	2 (± 0.3)	11 (± 1.8)	11 (± 2)	4 (± 0.6)	16 (± 2.4)
30 µM Tafenoquine	3 (± 0.4)	2 (± 0.2)	4 (± 0.2)	5 (± 0.7)	4 (± 0)	6 (± 0.7)	2 (± 0.2)	3 (± 0.3)	1 (± 0.1)	2 (± 0.2)
703 µM EDHB	1 (± 0)	1 (± 0)	2 (± 0.4)	1 (± 0)	1 (± 0)	2 (± 0.4)	2 (± 0.4)	1 (± 0.2)	2 (± 0)	2 (± 0)

AcrB mutant G141D_N282Y	LVX	MXF	LZD	CLI	OXA	CXM	NOV	MIN	RIX	AZM
MIC [µg/ml][b] drug alone	1 (± 0)	2 (± 0)	910 (± 41)	591 (± 36)	332 (± 23)	25 (± 2)	217 (± 24)	3 (± 0.3)	20 (± 3)	158 (± 19)
	**fold MIC decrease[b] with EPI[c]**									
48 µM PAβN	4 (± 0)	8 (± 0)	11 (± 2.2)	23 (± 7.6)	5 (± 1.1)	1 (± 0.3)	85 (± 17.4)	16 (± 0)	91 (± 30.5)	32 (± 0)
440 µM NMP	4 (± 0)	7 (± 0.7)	6 (± 0.7)	9 (± 1.1)	4 (± 0.5)	4 (± 0.6)	5 (± 0.5)	12 (± 1.6)	3 (± 0.3)	10 (± 1.7)
25 µM MBX2319	2 (± 0)	1 (± 0.3)	1 (± 0.3)	2 (± 0.3)	1 (± 0.3)	1 (± 0.3)	1 (± 0.1)	1 (± 0.4)	1 (± 0)	3 (± 0.5)
25 µM MBX2931	4 (± 0)	3 (± 0.5)	3 (± 0.5)	4 (± 0)	3 (± 0.5)	7 (± 1.1)	2 (± 0)	2 (± 0)	2 (± 0.3)	5 (± 1.4)
12.5 µM MBX3132	6 (± 0.9)	11 (± 4.7)	6 (± 2.1)	6 (± 0.9)	7 (± 1.1)	13 (± 1.8)	5 (± 0.7)	6 (± 2.4)	3 (± 0.4)	7 (± 1.1)
12.5 µM MBX3135	7 (± 0.9))	11 (± 6.1)	7 (± 2.6)	6 (± 1.3)	10 (± 1.7)	10 (± 1.7)	6 (± 1)	8 (± 2.4)	3 (± 0.5)	7 (± 0.9)
12.5 µM MBX3796	8 (± 0)	5 (± 1.1)	7 (± 1.1)	9 (± 2.9)	7 (± 1.1)	19 (± 5.8)	9 (± 2.9)	5 (± 1.1)	3 (± 0.5)	16 (± 0)
50 µM BM-19	2 (± 0)	4 (± 0)	6 (± 1)	4 (± 0)	2 (± 0)	2 (± 0)	4 (± 0.4)	6 (± 1)	10 (± 1.7)	2 (± 0.6)
50 µM Sertraline	2 (± 0.3)	2 (± 0)	2 (± 0)	3 (± 0.5)	2 (± 0)	3 (± 0.5)	1 (± 0.3)	2 (± 0.7)	2 (± 0.7)	1 (± 0.1)
165 µM NDGA	3 (± 0.7)	2 (± 0.4)	2 (± 0)	12 (± 2.8)	3 (± 0.7)	4 (± 0)	2 (± 0)	1 (± 0)	4 (± 0)	8 (± 0)
340 µM Proguanil	8 (± 0)	16 (0)	16 (± 0)	16 (± 0)	13 (± 2.2)	3 (± 0.5)	16 (± 0)	11 (± 2.2)	13 (± 2.2)	27 (± 4.4)
30 µM Tafenoquine	3 (± 0.5)	4 (± 0)	4 (± 0)	5 (± 1.5)	7 (± 0.9)	7 (± 1.3)	2 (± 0)	3 (± 0.5)	2 (± 0)	1 (± 0.2)
509 µM PCPP	4 (± 0)	5 (± 1.1)	3 (± 0.5)	3 (± 0.5)	3 (± 0.5)	2 (± 0)	2 (± 0)	7 (± 1.1)	1 (± 0.3)	4 (± 0)
100 µM BDM88855	12 (± 2.8)	16 (± 0)	32 (± 0)	8 (± 0)	24 (± 5.7)	32 (± 0)	16 (± 0)	12 (± 2.8)	4 (± 0)	32 (± 0)
50 µM NA #17	2 (± 0.3)	4 (± 1.6)	5 (± 1.4)	5 (± 1.1)	3 (± 0.5)	4 (± 0)	2 (± 0.3)	2 (± 0.3)	9 (± 2.9)	23 (± 7.6)
25 µM NA #18	1 (± 0.3)	1 (± 0)	5 (± 1.4)	3 (± 0.5)	1 (± 0)	3 (± 0.5)	1 (± 0)	2 (± 0)	4 (± 1.8)	9 (± 2.9)
50 µM NA #20	3 (± 0.5)	4 (± 0)	8 (± 0)	11 (± 2.2)	13 (± 2.2)	11 (± 2.2)	1 (± 0)	4 (± 0)	16 (± 0)	7 (± 1.1)

AcrB mutant V411A	LVX	MXF	LZD	CLI	OXA	CXM	NOV	MIN	RIX	AZM
MIC [µg/ml][b] drug alone	2 (± 0)	4 (± 0)	1024 (± 0)	819 (± 74)	512 (± 0)	30 (± 2)	307 (± 32)	2 (± 0.2)	19 (± 2)	230 (± 16)
	**fold MIC decrease[b] with EPI[c]**									
48 µM PAβN	4 (± 0)	8 (± 0)	16 (± 0)	8 (± 0)	6 (± 1.4)	1 (± 0)	96 (± 23)	8 (± 0)	24 (± 6)	12 (± 3)
440 µM NMP	8 (± 0)	8 (± 0)	32 (± 0)	9 (± 2.2)	4 (± 0)	5 (± 0.9)	4 (± 1.2)	8 (± 0)	2 (± 0.2)	7 (± 0.9)
25 µM MBX2319	8 (± 0)	4 (± 0)	4 (± 0)	5 (± 2.1)	8 (± 0)	6 (± 1.4)	2 (± 0.4)	6 (± 1.4)	2 (± 0.4)	6 (± 1.4)
25 µM MBX2931	12 (± 3)	24 (± 6)	16 (± 0)	12 (± 2.8)	24 (± 5.7)	24 (± 5.7)	10 (± 4)	12 (± 3)	3 (± 0.7)	48 (± 11)
12.5 µM MBX3132	32 (± 0)	32 (± 0)	64 (± 0)	64 (± 0)	128 (± 0)	96 (± 22.6)	80 (± 34)	16 (± 0)	6 (± 1.4)	64 (± 0)
12.5 µM MBX3135	16 (± 0)	32 (± 0)	48 (± 11)	64 (± 0)	192 (± 45)	128 (± 0)	64 (± 0)	16 (± 0)	4 (± 0)	48 (± 11)
12.5 µM MBX3796	16 (± 0)	32 (± 0)	48 (± 11)	48 (± 11)	96 (± 23)	96 (± 22.6)	80 (± 34)	16 (± 0)	6 (± 1.4)	48 (± 11)
50 µM BM-19	2 (± 0)	4 (± 0)	6 (± 1.4)	6 (± 1.4)	2 (± 0)	1 (± 0)	8 (± 0)	4 (± 0)	8 (± 0)	2 (± 0)
100 µM Sertraline	3 (± 0.7)	2 (± 0)	3 (± 0.7)	3 (± 0.7)	3 (± 0.7)	5 (± 2)	1 (± 0.2)	2 (± 0.4)	1 (± 0)	2 (± 0)
330 µM NDGA	2 (± 0)	2 (± 0.4)	2 (± 0)	8 (± 0)	4 (± 0)	4 (± 0)	2 (± 0.4)	2 (± 0.4)	3 (± 1)	2 (± 0)
340 µM Proguanil	4 (± 0)	8 (± 0)	16 (± 0)	16 (± 0)	8 (± 0)	2 (± 0)	8 (± 0)	12 (± 3)	4 (± 0)	16 (± 0)
30 µM Tafenoquine	2 (± 0)	2 (± 0)	3 (± 0.7)	6 (± 1.4)	4 (± 0)	4 (± 0)	1 (± 0)	2 (± 0)	1 (± 0)	2 (± 0)
509 µM PCPP	4 (± 0)	8 (± 0)	4 (± 0)	6 (± 1.4)	4 (± 0)	2 (± 0.4)	3 (± 0.7)	6 (± 1.4)	2 (± 0.4)	8 (± 0)
100 µM BDM88855	1 (± 0)	1 (± 0)	2 (± 0)	2 (± 0)	1 (± 0)	2 (± 0)	1 (± 0)	1 (± 0)	1 (± 0)	1 (± 0.2)
50 µM NA #17	8 (± 0)	2 (± 0)	16 (± 0)	4 (± 0)	8 (± 0)	8 (± 0)	ND	2 (± 0)	ND	ND
25 µM NA #18	1 (± 0)	2 (± 0.4)	8 (± 0)	2 (± 0)	1 (± 0)	2 (± 0.4)	1 (± 0)	3 (± 0.7)	16 (± 0)	24 (± 6)
50 µM NA #20	4 (± 0)	3 (± 0.7)	8 (± 0)	9 (± 5)	34 (± 21)	34 (± 21)	65 (± 45)	3 (± 0.7)	16 (± 0)	64 (± 0)

[a] LVX, levofloxacin; MXF, moxifloxacin, LZD, linezolid; CLI, clindamycin; OXA, oxacillin; CXM, cefuroxime; NOV, novobiocin; MIN, minocycline; RIX, rifaximine; AZM, azithromycin.

[b] Standard error given in parentheses. ND, not determined.

[c] fold decrease was calculated as quotient of the MIC without EPI and in the presence of EPI (mean values of ≥ 2 replicates shown). NDGA, nordihydroguaretic acid; PCPP, p-chlorophenylpiperazine, NA, naphthylamide EPI; EDHB, Ethyl 3,4-dihydroxybenzoate.

Table S2. Maximum distance change between selected residues during conformational cycling in RND-type transporters

RND-type transporter						Maximum distance change between residue pairs[a] [%]				
Entry ID[b]	Trans-porter	Bound substrates or inhibitors[c]	Mutations	Method	Resolution (Å)	I38, I671	G141, N282	G616, F610	F178, F628	F617, R717
2DRD	AcrB	Minocycline chain A (**DBP**)		X-RAY	3.1	**57.9**	8.7	**21.6**	**71.4**	**107.5**
2DHH	AcrB			X-RAY	2.8	**60.3**	6.7	**20.8**	**84.6**	**100.0**
2DR6	AcrB	Doxorubicin chain A (**DBP**)		X-RAY	3.3	**67.8**	4.8	7.8	**76.3**	**34.4**
2HRT	AcrB			X-RAY	3	**26.9**	0.9	9.2	**118.2**	**33.9**
2GIF	AcrB			X-RAY	2.9	**40.3**	5.6	4.6	**111.1**	**26.4**
2J8S	AcrB			X-RAY	2.54	**37.5**	0.9	**21.8**	**83.7**	**30.6**
3AOA	AcrB			X-RAY	3.35	**20.0**	9.5	**16.8**	**51.1**	**219.6**
3AOB	AcrB	Rifampicin chain C (**PBP**)		X-RAY	3.35	**46.9**	1.9	11.3	**36.4**	**52.7**
3AOD	AcrB	Minocycline chain A (**DBP**); Rifampicin chain C (**PBP**)		X-RAY	3.3	**78.9**	4.8	8.3	**45.2**	**37.3**
3AOC	AcrB	ERY chain C (**PBP**)		X-RAY	3.34	**91.7**	5.6	3.8	**23.4**	**65.3**
4DX6	AcrB		G616N	X-RAY	2.9	**17.1**	3.8	10.7	**102.3**	**21.7**
4DX5	AcrB	Minocycline chain B (DBP)		X-RAY	1.9	**31.3**	3.7	**16.8**	**123.8**	**15.7**
4DX7	AcrB	Doxorubicin chain B (**DBP**), chain A (2x **PBP**)		X-RAY	2.25	**36.2**	2.8	**16.7**	**121.4**	**17.4**
3W9H	AcrB	D13-9001 chain B (**DBP**)		X-RAY	3.5	**23.3**	3.7	7.4	**71.1**	**48.3**
4U8Y	AcrB	Minocycline chain B (**DBP**)	D408N	X-RAY	2.1	**31.8**	2.8	**15.1**	**131.0**	12.0
4U95	AcrB	Minocycline chain B (**DBP**)	K940A	X-RAY	2	**37.2**	2.8	**15.1**	**136.6**	11.0
4U8V	AcrB	Minocycline chain B (**DBP**)	D407N	X-RAY	2.3	**65.0**	2.8	**15.1**	**88.6**	**15.3**
4U96	AcrB		R971A	X-RAY	2.2	**43.2**	3.8	11.3	**100.0**	10.0
4ZIW	AcrB		Del F615-R620	X-RAY	3.4	**26.8**	7.6	1.7	**128.9**	12.2
4ZIT	AcrB			X-RAY	3.3	**33.3**	6.7	2.3	**116.3**	**21.4**
4ZIV	AcrB		F615A, F617A, R620A	X-RAY	3.16	**37.5**	4.6	3.0	**54.2**	**32.9**
4ZJL	AcrB	ERY chain A (**PBP**)		X-RAY	3.47	**46.3**	5.5	6.5	**122.2**	**76.1**
5JMN	AcrB	Fusidic acid chain A, B, C (**TM**)		X-RAY	2.5	**22.0**	2.8	**18.9**	**95.2**	**16.7**
5NC5	AcrB	Puromycin chain B (**DBP**)		X-RAY	3.2	**17.9**	3.7	**19.0**	**123.8**	**33.6**
5YIL	AcrB			X-RAY	3	**36.0**	1.9	8.3	**74.4**	**31.3**
6CSX	AcrB			CryoEM	3	**88.4**	3.7	1.6	6.8	**27.3**
6BAJ	AcrB			CryoEM	3.2	**81.6**	7.6	3.8	**22.0**	**28.4**
6Q4N	AcrB	Fusidic acid chain B (**TM**); fusidic acid chain C (**TM**)	V340N	X-RAY	2.8	6.4	3.8	**16.3**	**135.0**	**33.3**
6Q4P	AcrB	Fusidic acid chain B, chain C (**TM**)	N298A	X-RAY	2.8	**22.0**	5.7	**20.0**	**102.4**	**72.6**
6Q4O	AcrB	Fusidic acid chain B (**TM**)	I27A	X-RAY	2.8	**25.0**	4.8	13.8	**126.8**	**24.8**
6SGR	AcrB			CryoEM	3.17	3.9	1.8	4.6	**38.1**	**29.6**
6SGS	AcrB			CryoEM	3.2	12.2	1.8	7.0	**50.0**	**28.6**
6ZO8	AcrB	Minocycline chain B (**DBP**)	G621P	X-RAY	2.5	7.7	3.8	**14.7**	**119.0**	**57.4**
6ZOD	AcrB	Fusidic acid chain A (**TM**) & chain B (**TM**)		X-RAY	2.85	**15.7**	7.6	**15.7**	**107.7**	**60.3**
6ZO6	AcrB	Minocycline chain B (**DBP**)	G619P	X-RAY	2.35	**16.3**	4.8	**19.0**	**126.2**	**24.1**
6ZOB	AcrB	3-Formylrifamycin chain A (**PBP**)		X-RAY	2.8	**25.0**	5.7	**19.8**	**83.3**	**96.2**
6ZOC	AcrB	ERY chain A (**PBP**); 3-formylrifamycin chain B (**PBP**)	G616P	X-RAY	2.89	**30.4**	3.7	9.4	**142.5**	**116.2**
6ZO5	AcrB	Fusidic acid chain B (**TM**)	G619P, G621P	X-RAY	2.5	**33.3**	4.7	8.6	**79.1**	**92.9**
6ZOH	AcrB	3-Formylrifamycin chain B (**PBP**)	G619P, G621P	X-RAY	2.8	**35.0**	3.8	4.0	**88.1**	**158.3**
6ZO7	AcrB	3-Formylrifamycin chain A, chain B (**PBP**)	G619P	X-RAY	2.85	**38.1**	6.7	9.8	**87.5**	**85.5**
6ZOF	AcrB	Fusidic acid chain B (**TM**)	F380A	X-RAY	3.3	**38.6**	5.7	**17.3**	**114.6**	**28.8**
6ZOG	AcrB	Minocycline chain B (**DBP**)	I38F, I671T	X-RAY	2.75	**41.7**	4.8	12.4	**124.4**	**20.8**
6ZO9	AcrB	Rifabutin chain B (2x **PBP**)	G621P	X-RAY	2.7	**51.1**	5.7	11.5	**69.8**	**94.1**
6ZOA	AcrB			X-RAY	3.5	**136.2**	4.8	3.4	**105.0**	**19.2**
7OUL	AcrB	BDM88832 chain A (**TM**)	R971A	X-RAY	2.8	**31.0**	4.7	14.0	**100.0**	**52.3**
7OUM	AcrB	BDM88855 chain A (**TM**), chain B (**interprotom. loop**)	R971A	X-RAY	2.45	**33.3**	3.7	**15.6**	**90.5**	**34.7**
7OUK	AcrB	BDM88855 chain A (**TM**)		X-RAY	2.6	**46.3**	5.6	10.7	**88.1**	**50.0**
7KGE	AdeB			CryoEM	3.21	9.1	12.3	1.5	7.0	4.0
7KGG	AdeB	EtBr chain C (2x **DBP**, 1x **PBP**)		CryoEM	2.97	**26.4**	6.3	5.3	**24.3**	4.8
7M4P	AdeJ	Eravacycline chain B (**DBP**)		CryoEM	2.86	**20.5**	2.5	5.9	**40.7**	**43.1**
7M4Q	AdeJ			CryoEM	2.87	**77.4**	3.7	13.4	**31.1**	**79.2**
5LQ3	CmeB			X-RAY	3.55	**30.9**	3.5	9.3	**51.9**	**43.7**

5T0O	CmeB		X-RAY	3.15	8.0	3.7	4.3	**29.6**	11.6
2V50	MexB		X-RAY	3	**40.8**	3.6	12.7	**52.3**	**39.3**
3W9I	MexB		X-RAY	2.71	**12.8**	3.6	**22.1**	**104.9**	**21.4**
3W9J	MexB	D13-9001 chain B (**DBP**)	X-RAY	3.15	**54.5**	9.6	**16.1**	**52.3**	12.5
6IIA	MexB		X-RAY	2.91	10.9	4.6	**17.8**	**95.0**	**28.8**
6TA6	MexB		CryoEM	3.2	3.4	**15.2**	5.8	**42.6**	14.7
6VKT	MtrD	ERY chain B (**DBP**)	CryoEM	2.72	3.2	5.2	7.0	**45.2**	8.1
6VKS	MtrD	Ampicillin chain C (**DBP**)	CryoEM	3.2	**15.0**	11.6	4.2	**19.2**	12.3
7CZ9	OqxB		X-RAY	1.85	7.6	1.1	0.6	2.5	1.1

[a] Homologous residues in RND transporters others than AcrB (Table S3) determined by multiple alignments using Clustal Omega (Clustal Omega Multiple Sequence Alignment EMBL-EBI) (1)). Distances between residues of the indicated pairs were measured in chain A, B and C using PyMOL (2) and the difference between the maximum and the minimum distance was determined. The maximum percent change was calculated relative to the minimum distance (changes ≥ 15 % highlighted in bold). Measurement details given in table S3.

[b] Structures from the RCSB Protein Data Bank (RCSB PDB) (3).

[c] Binding regions of ligands (substrates or inhibitors) are indicated bold-faced (DBP, distal binding pocket; PBP, proximal binding pocket; TM, transmembrane domain region); ERY, erythromycin; BDM88832, 1-(3-chloro-5-iodo-2-pyridyl)piperazine; BDM88855, 3-chloro-2-piperazin-1-yl-quinoline.

Table S3. Atoms of amino acid residues used for distance measurements in RND transporters.

Transporter	Residue 1[a]	Atom[b] residue 1	Residue 2[a]	Atom[b] residue 2
**AcrB**				
	I38	C4	I671	C4
	G141	C2	N282	C4
	G616	C2	F610	C7
	F178	C7	F628	C7
	R717	C7	F617	C7
**AdeB**				
	V38	C3	I663	C4
	G141	C2	N282	C4
	G611	C2	T605	C3
	F178	C7	F623	C7
	W708	C8	F612	C7
**AdeJ**				
	I38	C4	M673	S5
	A141	C3	N282	C4
	S617	C3	F611	C7
	F178	C7	F629	C7
	R718	C7	F618	C7
**CmeB**				
	L39	C4	I666	C4
	S142	C3	N284	C4
	D612	C4	M606	S5
	I179	C4	F626	C7
	R711	C7	L613	C4
**MexB**				
	I38	C4	V671	C3
	G141	C2	N282	C4
	N616	C4	F610	C7
	F178	C7	F628	C7
	R716	C7	F617	C7
**MtrD**				
	V38	C3	I667	C4
	M141	C4	N280	C4
	S611	C3	I605	C4
	F176	C7	F623	C7
	R714	C7	F612	C7
**OqxB**				
	V40	C3	I676	C4
	H143	C4	N285	C4
	N622	C4	V616	C3
	F180	C7	F636	C7
	I721	C4	A623	C3

[a] AcrB residues and respective homologs (alignments done with Clustal Omega (1), see table S2).

[b] Atom-numbering according to the IUPAC nomenclature.

Table S4 HPLC-MS parameters.

HPLC-MS device	LC - 20AD from Shimadzu (Kyoto, Japan) with a Sciex Triple Quad™ 4500 (Toronto, Canada)
Column	Unison UK-C18, 3 µm, 50 x 2 mm from Imtakt (Kyoto, Japan)
Run time	3 min
Gradient phases:<br>Mobile phase A<br>Mobile phase B	 0.1% formic acid in H_2O (v/v)<br>0.1% formic acid in MeOH (v/v)
Gradient	Starting with 95% of phase A until 12 sec, gradually switched to 90% of B until 66 sec. This was kept until 108 sec and then gradually switched back to 95% of A. Re-equilibration started at 150 sec and lasted until the run time of 180 sec was reached.
Autosampler temperature	Held at 4 (+/-1) °C
Column oven temperature	Set to 40 °C
MS operating mode	Multiple reaction monitoring (MRM) with negative electrospray ionization (ESI) mode

MS parameters for linezolid and oxacillin

(one transition used for quantification (quantifier), the others to confirm the identity of the ion (qualifiers).

		Mother ion (m/z)	Daughter ion (m/z)	RT[a] (min)	DP[b] (V)	CE[c] (V)	CXP[d] (V)
Linezolid 1	Quantifier	337.600	296.000	2.05	106	25	4
Linezolid 2	Qualifier	337.600	235.000	2.05	106	29	4
Linezolid 3	Qualifier	337.600	148.000	2.05	96	51	10
Linezolid 4	Qualifier	337.600	135.000	2.05	96	70	12
Oxacillin 1	Quantifier	401.900	160.000	2.25	61	19	10
Oxacillin 2	Qualifier	401.900	243.000	2.25	61	19	10

[a] RT, retention time.

[b] DP, declustering potential.

[c] CE, collision energy.

[d] CXP, collision cell exit potential.

Fig. S1

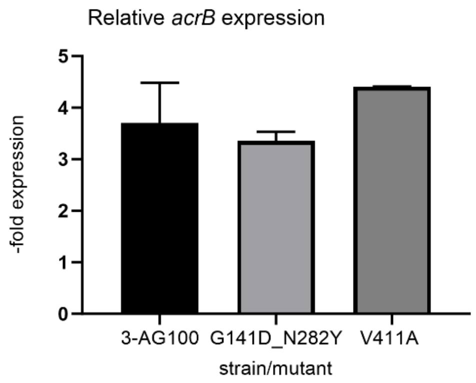

Fig S1. Expression of *acrB* relative to that of the housekeeping gene *gyrB* determined by quantitative real-time reverse transcription PCR (qRT-PCR). Means of two independent experiments with SEM are shown. No statistically significant differences (P <0.05) were detectable between the *acrB* expression values of the strains. The qRT-PCR experiments had been carried out according to procedures described previously (4) by using the following primer pairs: *acrB*, 5'-gaacaa-ctggcgagcaaact-3' and 5'-gcataacggagaacggaatc-3'; *gyrB*, 5'-cagtacattaaa-gacgacgaagc-3' and 5'-atcattttctgcgtcgcgtt-3'.

Fig. S2

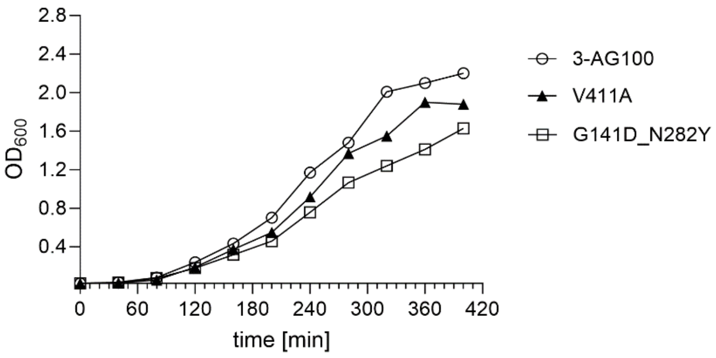

Fig. S2. Growth curves of the parental *E. coli* 3-AG100 and AcrB mutants. The doubling time in the exponential phase was 54 min for the wild-type AcrB strain 3-AG100, 60 min for the double-mutant, and 58 min for the V411A mutant (means calculated from three experiments).

REFERENCES

1. Sievers F, Wilm A, Dineen D, Gibson TJ, Karplus K, Li W, Lopez R, McWilliam H, Remmert M, Soding J, Thompson JD, Higgins DG. 2011. Fast, scalable generation of high-quality protein multiple sequence alignments using Clustal Omega. *Mol Syst Biol* **7**:539. https://doi.org/10.1038/msb.2011.75.
2. The PyMOL Molecular Graphics System, Version 2.5.1, Schrödinger, LCC.
3. Berman HM, Westbrook J, Feng Z, Gilliland G, Bhat TN, Weissig H, Shindyalov IN, Bourne PE. The Protein Data Bank. 2000. *Nucleic Acids Research* **28**: 235-242. https://doi.org/10.1093/nar/28.1.235.
4. Schuster S, Vavra M, Köser R, Rossen JWA, Kern WV. 2021. New topoisomerase inhibitors: Evaluating the potency of gepotidacin and zoliflodacin in fluoroquinolone-resistant *Escherichia coli* upon *tolC* inactivation and differentiating their efflux pump substrate nature. *Antimicrob Agents Chemother* **65**:AAC.01803-20 [pii];10.1128/AAC.01803-20 [doi].
